# Rapid Detection of Antimicrobial Resistance in Mycoplasma genitalium by High-Resolution Melting Analysis with Unlabeled Probes

**DOI:** 10.1128/spectrum.01014-22

**Published:** 2022-07-26

**Authors:** Yamei Li, Leshan Xiu, Liqin Wang, Lulu Zhang, Feng Wang, Junping Peng

**Affiliations:** a NHC Key Laboratory of Systems Biology of Pathogens, Institute of Pathogen Biology, Chinese Academy of Medical Sciences and Peking Union Medical College, Beijing, China; b Key Laboratory of Respiratory Disease Pathogenomics, Chinese Academy of Medical Sciences, Beijing, China; c School of Global Health, Chinese Center for Tropical Diseases Research, Shanghai Jiao Tong University School of Medicine, Shanghai, China; d One Health Center, Shanghai Jiao Tong University-The University of Edinburgh, Shanghai, China; e Shenzhen Center for Chronic Disease Control, Shenzhen, China; University of Utah and ARUP Laboratories

**Keywords:** antimicrobial resistance, *Mycoplasma genitalium*, unlabeled probe, high-resolution melting technology

## Abstract

With looming resistance to fluoroquinolones in Mycoplasma genitalium, public health control strategies require effective antimicrobial resistance (AMR) diagnostic methods for clinical and phenotypic AMR surveillance. We developed a novel AMR detection method, MG*parC*-AsyHRM, based on the combination of asymmetric high-resolution melting (HRM) technology and unlabeled probes, which simultaneously performs M. genitalium identification and genotypes eight mutations in the *parC* gene that are responsible for most cases of fluoroquinolone resistance. These enhancements expand the traditional HRM from the conventional detection of single-position mutations to a method capable of detecting short fragments with closely located AMR positions with a high diversity of mutations. Based on the results of clinical sample testing, this method produces an accordance of 98.7% with the Sanger sequencing method. Furthermore, the specificity for detecting S83I, S83N, S83R, and D87Y variants, the most frequently detected mutations in fluoroquinolone resistance, was 100%. This method maintained a stable and accurate performance for genomic copies at rates of ≥20 copies per reaction, demonstrating high sensitivity. Additionally, no specific cross-reactions were observed when testing eight common sexually transmitted infection (STI)-related agents. Notably, this work highlights the significant potential of our method in the field of AMR testing, with the results suggesting that our method can be applied in a range of scenarios and to additional pathogens. In summary, our method enables high throughput, provides excellent specificity and sensitivity, and is cost-effective, suggesting that this method can be used to rapidly monitor the molecular AMR status and complement current AMR surveillance.

**IMPORTANCE** Mycoplasma genitalium was recently added to the antimicrobial-resistant (AMR) threats “watch list” of the U.S. Centers for Disease Control and Prevention because this pathogen has become extremely difficult to treat as a result of increased resistance. M. genitalium is also difficult to culture, and therefore, molecule detection is the only method available for AMR testing. In this work, we developed a novel AMR detection method, MG*parC*-AsyHRM, based on the combination of asymmetrical HRM technology and unlabeled probes, and it simultaneously performs M. genitalium identification and genotypes eight mutations in the *parC* gene that are responsible for most cases of fluoroquinolone resistance. The MG*parC*-AsyHRM method is a high-throughput, low-cost, simple, and culture-free procedure that can enhance public health and management of M. genitalium infections and AMR control, providing a strong complement to phenotypic AMR surveillance to address the spread of fluoroquinolone resistance.

## INTRODUCTION

Mycoplasma genitalium is an important sexually transmitted bacterium responsible for 10% to 35% of cases of nongonococcal urethritis in men and has been associated with cervicitis and pelvic inflammatory disease in women (1, 2). International guidelines recommend macrolide (azithromycin) and fluoroquinolone (moxifloxacin) antibiotics as the first- and second-line treatments, respectively (3, 4). In many countries, more than 50% of cases are macrolide resistant but are successfully treated with fluoroquinolones (5). However, over the past decade, there has been an increase in resistance to both classes of drugs when treating M. genitalium infections, to the extent that it is becoming a global public health concern (6–8). The prevalence of fluoroquinolone resistance in M. genitalium samples is rapidly increasing in the World Health Organization Western Pacific Region (6). As there are few alternative treatments available for M. genitalium, any strategy that prolongs the effectiveness of existing treatments, especially fluoroquinolones, should be considered. Therefore, rapid and sensitive detection of fluoroquinolone resistance in M. genitalium is needed to monitor antimicrobial susceptibility and maintain the effectiveness of current treatment regimens.

Antimicrobial susceptibility testing is generally performed using culture-based methods, which are highly specific but challenging to implement due to the difficulty in culturing M. genitalium (9). Large-scale screening studies and surveillance programs can identify many underlying mutations associated with antimicrobial resistance (AMR) (6–8), which has made it possible to develop molecular methods for screening genetic markers of AMR in M. genitalium. The primary mutations associated with fluoroquinolone resistance in M. genitalium are amino acid changes at positions 83 and 87 (including S83C, S83I, S83N, S83R, D87G, D87H, D87N, and D87Y) of the *parC* gene, and they are associated with treatment failure and elevated MIC *in vitro* test results for fluoroquinolones (7, 10). Accordingly, a molecular assay capable of exhaustively detecting amino acid changes in *parC* could predict fluoroquinolone resistance with high sensitivity and specificity and could facilitate efforts to control the spread of resistant isolates and ensure pathogen eradication. In particular, a rapid molecular test to distinguish the wild-type and S83I mutation could be extremely useful in clinical practice because the S83I mutation is considered a potential predictive marker in patient management across many parts of the world (11).

M. genitalium is extremely difficult to culture, and therefore, nucleic acid amplification testing (NAAT) is the only method available for AMR testing of clinical specimens of M. genitalium. Several molecular methods have recently been developed to improve laboratory diagnostics of M. genitalium infection, as well as to address the need for resistance detection (12–16). Unfortunately, the high diversity and close proximity of mutations (see Fig. S1 in the supplemental material) pose a significant challenge for developing a comprehensive M. genitalium AMR diagnostic method. Conventional PCR and sequencing provide high sensitivity and specificity but require the PCR product to be evaluated using gel electrophoresis (17). Real-time PCR (RT-PCR) is the most popular method for detecting AMR-conferring mutations in M. genitalium. However, in order to detect multiple mutations within short sequences, multiple labeled probes are required for a single diagnostic assay. This inherent limitation of RT-PCR increases the cost and time required for each assay, challenges instrument capabilities, and requires more complex reaction conditions (13–16). Ideally, molecular diagnostics for the AMR of M. genitalium would use whole-genome sequencing (WGS) to effectively identify all known and potentially new genes and mutations that can predict both the AMRs and the MICs of antimicrobials (18) against M. genitalium. However, WGS still has considerable costs and is technically demanding. In addition, M. genitalium is often a low-load infection; thus, achieving a good depth of coverage in WGS approaches is also very challenging, which limits their implementation in clinical practice.

Here, we propose a culture-free method (MG*parC-*AsyHRM) that can rapidly and consistently identify M. genitalium and mutations associated with fluoroquinolone resistance with no sequencing analysis step, thereby reducing the cost and time requirements associated with the method. This novel method is based on a high-resolution melting (HRM) analysis with unlabeled probes and complements current M. genitalium detection using RT-PCR and WGS. HRM is a convenient, closed-tube, and cost-efficient method that is widely used in several research fields, including variant scanning, species identification, and molecular typing (19–22). Although HRM is superior to RT-PCR for identifying many small insertions or deletions and complex mutations, differentiating between two or more possible single nucleotide polymorphisms at a site can be problematic when probes are not used (23, 24). Therefore, our assay integrates an unlabeled probe and multiplex asymmetric PCR with HRM analysis to rapidly detect bacteria and simultaneously identify eight types of mutations in *parC*, facilitating a comprehensive diagnosis of M. genitalium in a single-tube reaction. This method uses a small probe to address the challenge of detecting complex mutations. A smaller probe produces larger temperature differences from relatively few base mismatches within a short sequence (25). To produce the desired HRM products, the 3′ end of the unlabeled probe was blocked to prevent extension, and asymmetric PCR was used to produce excess complementary strands for the unlabeled probe. The probes for complex sequences with various mutations in multiple positions in *parC* were designed as degenerate codons for all mutation positions, except the most important variants, in order to improve matching despite the multiple variants. In addition, we further explored the feasibility of MG*parC-*AsyHRM by using different scenarios to provide foundational data for its application in AMR detection of other pathogens.

## RESULTS

### Description of the MG*parC*-AsyHRM method.

**FIG 1:**
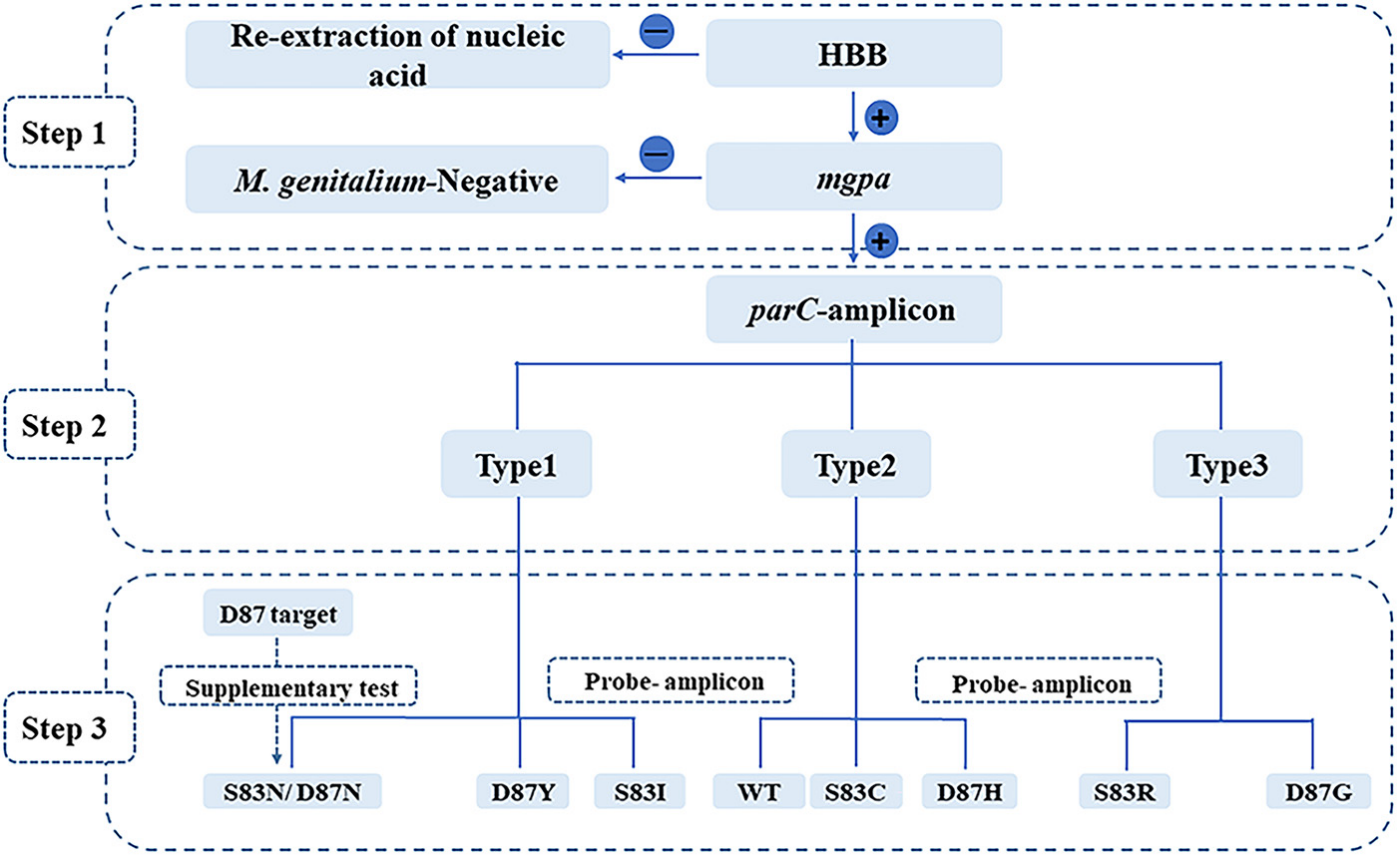
Workflow of the MG*parC*-AsyHRM method. WT, wild type.

The MG*parC*-AsyHRM method accurately distinguished eight types of mutations from the wild type using a combined melting temperature (*T_m_*) value from two amplicons. All primers were evaluated for uniqueness using the Basic Local Alignment Search Tool (BLAST) (https://www.ncbi.nlm.nih.gov/tools/primer-blast/) and further tested for accuracy by testing the known sequence plasmids (ParC wild type [WT], S83I, S83R, S83N, S83C, D87Y, D87N, D87G, and D87H). The optimal reaction conditions and primers are listed in Table 1. Because both the forward primer and probe were competitively binding to the reverse strand, the concentration difference between the probe and forward primer increased 26-fold. The method was divided into three major steps, as shown in Fig. 1. In the first step, all of the samples were tested to confirm that they were M. genitalium-positive and that the nucleic acids were successfully extracted by assay 1. In the second step, the main product type was determined through the *parC*-amplicon peak using assay 2. In the third step, the *parC* allele genotyping was performed by melting the probe-amplicon (Fig. 1). Notably, because the probe perfectly complemented the S83I sequence, the S83I variant showed a unique peak shape with the highest probe *T_m_*, indicating that the S83I variant could be detected quickly and directly.

**TABLE 1:** Optimal reaction conditions and primers used in this study

Assay	Target gene	Primer sequence	Concn (μM)	Significance
1	*mgpa*	MGpa_F, CTTGAGCCTTTCTAACCGCTGCACT	0.25	Species identification
		MGpa_R, CAAGTCCAAGGGGTTAAGGTTTCAT	0.25	Species identification
	*HBB*	HBB_F, AGTGCTCGGTGCCTTTAGTGAT	0.2	Quality control of nucleic acid extraction
		HBB_R, TGGCAAAGGTGCCCTTGA	0.2	Quality control of nucleic acid extraction
	*parC*	ParC_D87_F, CCCATGGTGATAGTTCCATTTAT	0.5	Supplementary test for distinguishing mutation S83N from D87N
		ParC_D87_R, AGCTTTGGGACATTCTGATAATTG	0.5	Supplementary test for distinguishing mutation S83N from D87N
2	*parC*	ParC_S8_F, GGGAGATCATGGGGAAATACC	0.0375	Prediction of fluoroquinolone resistance
		ParC_S83_R, CAGCTTTGGGACATTCTGATA	0.025	Prediction of fluoroquinolone resistance
		ParC_S83_P, CCCCCATGGTGATATTTCCATTTATDRTGCAA^*a*^	1	Prediction of fluoroquinolone resistance

a 3′-blocked oligonucleotide probe.

### Performance of the MG*parC*-AsyHRM method.

**FIG 2:**
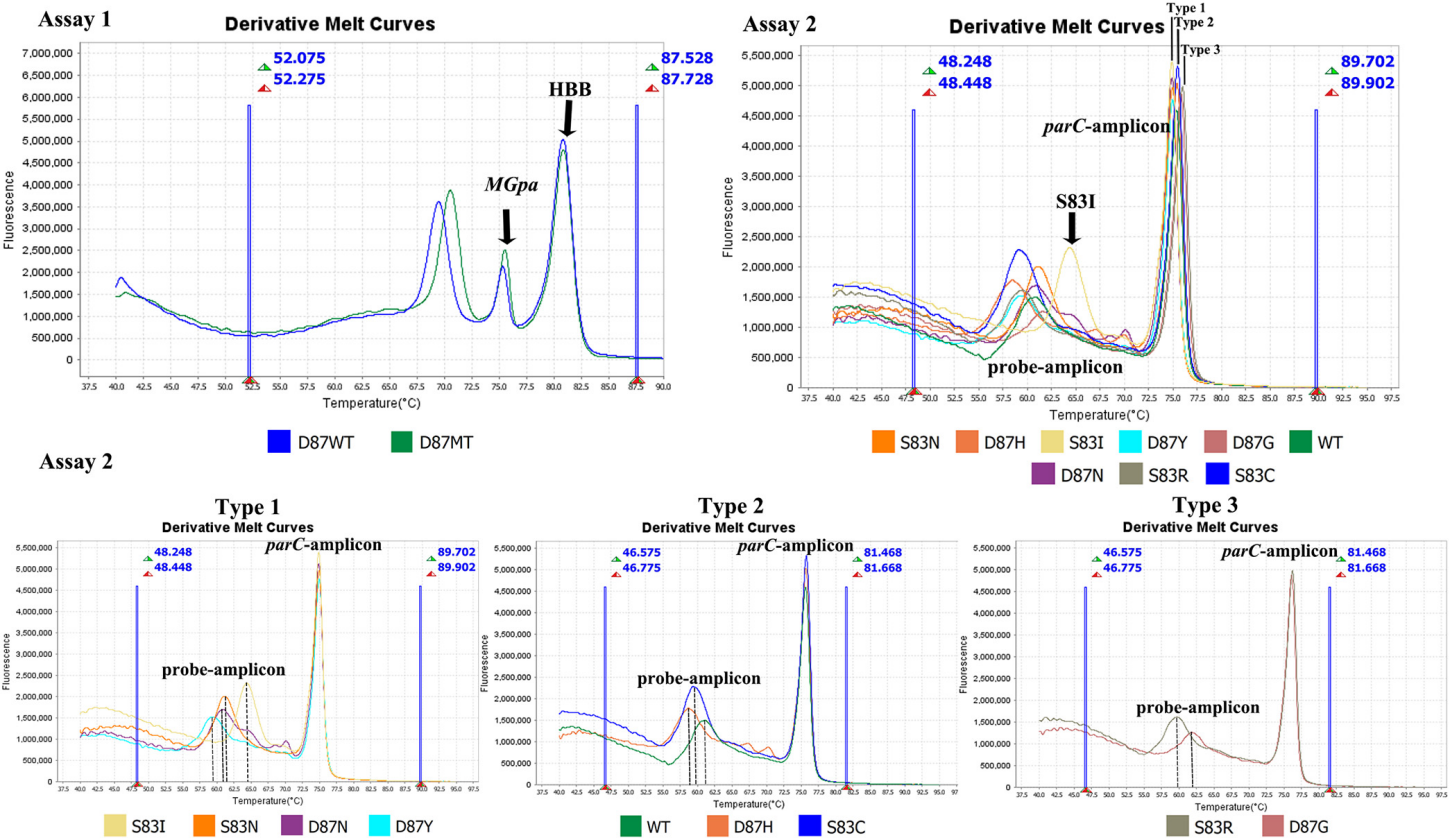
Results of assays 1 and 2 of the MG*parC*-AsyHRM method.

The known sequence plasmids (ParC WT, S83I, S83R, S83N, S83C, D87Y, D87N, D87G, and D87H) were accurately genotyped by the MG*parC*-AsyHRM method (Fig. 2). Furthermore, 9 plasmids containing various *parC* alleles were tested repeatedly at least 12 times to obtain stable *T_m_* ranges. The *T_m_* value of all variants is shown in Table 2. Based on these results, each variant was assigned a unique peak combination representing peaks for one *parC*-amplicon and one probe-amplicon.

**TABLE 2:** *T_m_* values for all variants

Assay	Target	PCR-amplicon type	Change	*T_m_* of:			
				PCR-amplicon		Probe-amplicon	
				Range	Mean ± SD	Range	Mean ± SD
1	*HBB*	Quality control of nucleic acid extraction		80.82–80.90	80.85 ± 0.024		
	*mgpa*	Species identification		75.44–75.67	75.60 ± 0.061		
	*parC*	D87WT		70.85–70.83	70.77 ± 0.068		
		D87MT		71.20–71.29	71.25 ± 0.027		
2	*parC*	Type 1	S83I (G248T)	75.05–75.20	75.10 ± 0.042	64.57–64.66	64.61 ± 0.027
			S83N (G248A)	75.03–75.13	75.10 ± 0.032	61.20–61.40	61.25 ± 0.008
			D87N (G259A)	75.01–75.13	75.17 ± 0.039	60.88–61.16	61.04 ± 0.067
			D87Y (G259T)	75.15–75.20	75.18 ± 0.022	60.24–60.39	60.32 ± 0.067
		Type 2	WT	75.57–75.71	75.60 ± 0.035	60.92–61.12	61.04 ± 0.056
			S83C (A247T)	75.65–75.76	75.70 ± 0.034	59.32–59.55	59.41 ± 0.073
			D87H (G259C)	75.53–75.65	75.60 ± 0.035	58.41–58.49	58.47 ± 0.037
		Type 3	S83R (A247C)	76.19–76.29	76.25 ± 0.030	59.36–59.52	59.42 ± 0.060
			D87G (A260G)	76.16–76.26	76.23 ± 0.024	62.14–62.41	62.25 ± 0.067

In the evaluation phase with the plasmid, assay 1 showed perfect sensitivity at 10 copies per reaction. Similarly, the *parC*-amplicon target showed the same limit of detection (LOD); however, the LOD of the probe-amplicon target was slightly higher but was still maintained at 20 copies per reaction (Table S3). The LOD of the probe-amplicon targets for common mutations was maintained at 10 copies per reaction (WT, S83N, S83I, S83R, D87Y, and D87N) (Table S3) (7). Considering the application scenario of this method, we performed a preliminary evaluation of the sensitivity of clinical samples, with the results shown in Table 3. Both assays showed a low success rate (assay 1, 74.1%; assay 2, 70%) at less than 20 genomic copies per reaction, which indicates that this method is not suitable for low-LOD clinical samples. In contrast, all other samples at various concentrations (≥20 copies per reaction) showed a high success rate (77/78, 98.7%). In general, the MG*parC*-AsyHRM assay showed stable performance when there were >20 genomic copies per reaction, and the fluorescent peak decreased slightly according to M. genitalium load. Based on the published results for infection loads of M. genitalium in clinical samples, our method can serve as an ideal tool for clinical antimicrobial stewardship among symptomatic populations (26).

**TABLE 3:** Performance of the MG*parC*-AsyHRM method with 105 fully characterized M. genitalium clinical samples

AMR position (no. of samples)	Consistency with Sanger sequence method (%)	No. of samples that failed to provide valid data/total no. of samples (%)	No. of samples successfully producing valid data for different genomic copy ranges/total no. of samples (%) with:							
			>2,000 copies/reaction		200–2,000 copies/reaction		20–200 copies/reaction		<20 copies/reaction	
			Assay 1	Assay 2	Assay 1	Assay 2	Assay 1	Assay 2	Assay 1	Assay 2
WT (31)	28/29 (96.6)	2/31 (6.5)	3/3 (100)	3/3 (100)	5/5 (100)	5/5 (100)	18/18 (100)	17/18 (94.4)	5/5 (100)	4/5 (80)
S83I (50)	43/43 (100)	7/50 (14)	6/6 (100)	6/6 (100)	11/11 (100)	11/11 (100)	20/20 (100)	20/20 (100)	9/13 (69.2)	9/13 (69.2)
S83N (11)	10/10 (100)	1/11 (9.1)	1/1 (100)	1/1 (100)	1/1 (100)	1/1 (100)	4/4 (100)	4/4 (100)	4/5 (80)	4/5 (80)
S83R (1)	1/1 (100)	0/1 (0)	0/0 (100)	0/0 (100)	0/0 (100)	0/0 (100)	1/1 (100)	1/1 (100)	0/0 (100)	0/0 (100)
D87N (7)	5/6 (83.3)	1/7 (14.3)	0/0 (100)	0/0 (100)	0/0 (100)	0/0 (100)	3/3 (100)	3/3 (100)	2/3 (66.7)	2/3 (66.7)
D87Y (4)	3/3 (100)	1/4 (25)	0/0 (100)	0/0 (100)	1/1 (100)	1/1 (100)	2/2 (100)	2/2 (100)	0/1 (0)	0/1 (0)
Rare type (1)	1/1 (100)	0/1 (0)	0/0 (100)	0/0 (100)	1/1 (100)	1/1 (100)	0/0 (100)	0/0 (100)	0/0 (100)	0/0 (100)
Total (*n* = 105)	91/93 (97.8)	12/105 (11.4)	11/11 (100)	11/11 (100)	19/19 (100)	19/19 (100)	48/48 (100)	47/48 (97.9)	20/27 (74.1)	19/27 (70)

All 105 control samples were confirmed as being M. genitalium positive by using RT-PCR. The distribution of the genomic copy number and the genotype of all samples are listed in Table 3. All samples were also shown to be human β-globin (*HBB*) positive, indicating that nucleic acid extraction was successful. Upon further testing, 12 samples (12/105, 11.4%) failed to provide comprehensive data (at least one assay failed to provide effective data), of which 11 samples (11/12, 91.7%) were due to low genomic copies (<20 copies per reaction). In addition, all failed samples were swab clinical samples. Among the remaining samples that provided valid AMR profiles (*n* = 93), the MG*parC*-AsyHRM method showed high agreement with the Sanger sequencing method (91/93, 97.9%). In clinical samples with ≥20 genomic copies per reaction, this method produced 98.7% (77/78) agreement with the Sanger sequencing method. The consistency with the Sanger sequencing method for S83I, S83N, S83R, and D87Y variants was 100%. The *mgpa* gene, used for species identification, produced a 91.42% (96/105) accordance based on RT-PCR. Of the samples that failed this test, 66.67% (6/9) failed due to a low LOD (<20 copies per reaction).

Additionally, one sample appeared to have a unique peak that did not belong to any known variant. According to the sequencing data, this sample was assigned as a rare mutation (G81C amino acid mutation), which indicates that our method can capture emerging mutations that are not involved in our assay in resistant transmission. In a two-way blind assessment, all 184 samples were assigned as M. genitalium positive or M. genitalium negative (4/184 positive, 180/184 negative), which was consistent with the results of the *mgpa* testing performed in assay 1. All samples were *HBB* positive. In addition, the four M. genitalium-positive samples showed 100% consistency between the AMR profiles and the Sanger sequencing method.

Finally, 33/33 (100%) sexually transmitted infection (STI)-related pathogens were tested individually and jointly. None of these tests showed any cross-reaction with the *mgpa* gene and *parC* probe, which suggests that this method could also be a useful pretest tool for clinical samples from patients with a coinfection.

### Flexibility of the MG*parC*-AsyHRM method.

**FIG 3:**
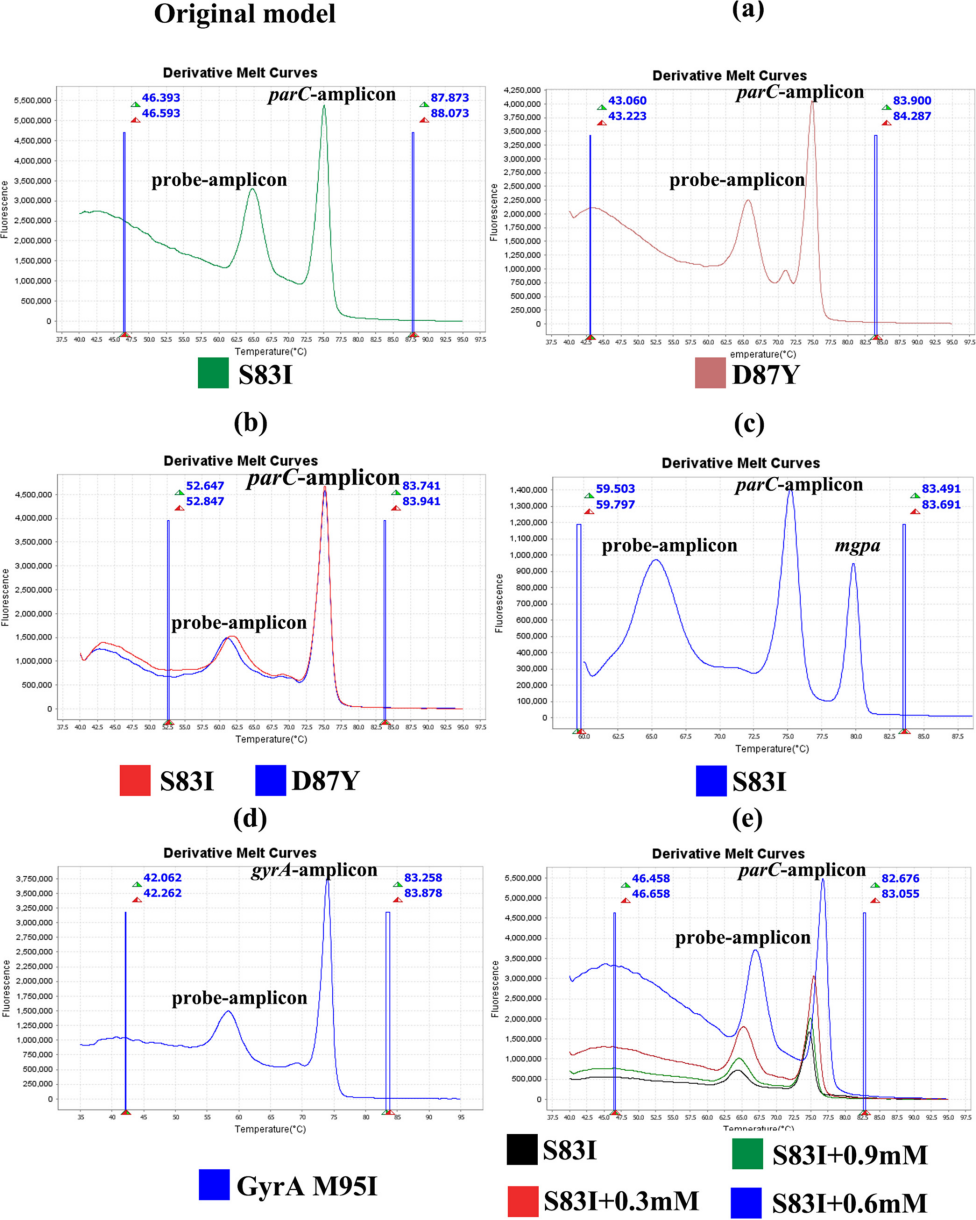
Flexibility of the MG*parC*-AsyHRM method. (a) Effect of a different probe. (b) Performance of a probe harboring double mutations (S83I plus D87Y). (c) Compatibility of the probe with other genes. (d) Generalizability of the MG*parC*-AsyHRM model. (e) Adjustability of the MG*parC*-AsyHRM model.

We further investigated the flexibility of this method to provide foundational data for the application of the MG*parC*-AsyHRM method to other pathogens. Using the S83I variant, we conducted preliminary exploration in five directions (Fig. 3). The results shown in Fig. 3a and 3b indicate that the probe can be applied to different mutations; however, the shape of the melting curve of each variant becomes smooth with additional mutations in the probe design. Therefore, it is necessary to select the most important mutation for designing the probe sequence to ensure the optimal performance of the method. As shown in Fig. 3c, the probe remained stable when coexisting with the *mgpa* gene, which indicates the possibility of single assay detection. For culturable pathogens, this method can be transformed into a single assay, which is particularly suitable for low-resource settings due to the low cost and high resolution of this method. To exclude gene preference, we performed additional testing using the *gyrA* gene, which may also lead to fluoroquinolone resistance in M. genitalium. The results shown in Fig. 3d demonstrate the generalizability of the MG*parC*-AsyHRM model with different AMR genes, using the *gyrA* gene as an example. Additionally, the *T_m_* value can be adjusted by adding Mg^2+^ to the reaction, which provides the possibility of adjusting the *T_m_* values of different amplicons to form multiple probe combinations (Fig. 3e). The optimal ion still needs to be further explored, as the ion concentration will affect both the temperature and the height of the peak.

## DISCUSSION

The spread of M. genitalium showing resistance to recommended antimicrobials remains a global concern, making it a potential “superbug” (27, 28). Because of increasing macrolide resistance, fluoroquinolones, as the second-line recommended therapy for M. genitalium infections, play a key role in many settings (3, 5). Thus, routinely available AMR detection for fluoroquinolone resistance is urgently needed. The aim of this study was to combine unlabeled oligonucleotide probes with HRM technology for identifying AMR mutations in clinical samples, including complex mutations, and to further establish a novel NAAT diagnostic method for identifying fluoroquinolone resistance in M. genitalium. There are two main reasons for choosing M. genitalium as the model pathogen in the process of establishing this new method. First, the isolation of M. genitalium from clinical specimens is exceedingly difficult, and NAATs are the only useful methods for species identification and AMR determination in M. genitalium (29). Consequently, M. genitalium is a high priority for the development of novel AMR diagnosis methods. Second, the mutations in the *parC* gene are closely linked to fluoroquinolone resistance, especially at amino acid positions S83 and D87 (8, 11). Notably, the sequence from S83 to D87 shows high diversity, with eight mutations across the two positions, which poses significant challenges for method design (see Fig. S1 in the supplemental material). These complicated sequences also allowed us to deeply explore the feasibility of this method.

Based on considerable preliminary evaluation, we have formed important recommendations for applying the MG*parC*-AsyHRM method. For AMR genes with mutations concentrated in a single amino acid site, such as the cephalosporin-related resistance mutation in the *penA* gene of Neisseria gonorrhoeae (P551S/L/T) (30), the recommended length of the probe is the same as the length commonly used in RT-PCR. The probe should be perfectly matched to the most important mutation without any degenerate codons, and the concentration difference of asymmetric amplification was referenced from a previous report (31). For short sequences containing multiple mutations in closely spaced AMR positions (such as rifampicin-related resistance mutations in the *rpoB* gene in Mycobacterium tuberculosis at positions 516, 526, and 531) (32), we suggest that the optimal length of the probe is in excess of 30 bp, whereas a probe that is too long or too short will affect the peak shape and diminish the resolution of this method. In addition, the probe should be perfectly matched to the most important mutation to maximize the advantages of this method, and the position of the mutation should be in the middle of the probe sequence. In the case of multiple mutation positions in the probe sequence, we suggest that all positions are designed as degenerate codons, except for the position corresponding to the most important mutation. However, each probe should have no more than two degenerate codons because the degenerate codons can weaken the mismatch of the probe and alter the shape of the melting peak. Additionally, to manage the dilution of probes caused by degenerate codons, we recommend increasing the concentration difference between the forward primer and probe up to 26-fold, with the concentration of the probe up to 1 μM instead of the routine 0.5 μM (31).

In recent decades, because of the complex environment of clinical samples, the nested PCR method has been widely used for clinical diagnosis and phenotypic AMR surveillance in M. genitalium instead of the standard PCR method (10, 33). However, less expensive, more effective, and easier-to-implement AMR diagnostic methods are needed to prevent M. genitalium infections and for epidemiological surveillance. Unlike macrolide resistance, there are few diagnostic methods available to test for fluoroquinolone resistance in M. genitalium (13, 34, 35). Previously, Tickner et al. utilized dual-hybridization probe assays to enable the detection of WT *parC* sequences that are strongly related to fluoroquinolone susceptibility. The introduction of fluorescent labeling further improves the flexibility of the method. Although both articles are based on the melting curve of the probe, our probes do not require a fluorescent label, which greatly reduces the detection cost. Compared to the dual-hybridization probe method, our method not only significantly improves sensitivity but also covers a more comprehensive mutation detection range (36). Thus, the development of our method fills this gap and provides a model for the diagnosis of AMR in other pathogens. Compared to previous methods, to the best of our knowledge, our method is the first to identify all mutations associated with fluoroquinolone resistance (10). This method can quickly and accurately genotype nine variants of the *parC* gene using unlabeled probes, as well as being high throughput, simple, and low cost. As the probe in this method does not contain a fluorescent label, the cost is as low as $3 per sample, which is far lower than that of other RT-PCR methods (10, 37). Additionally, the MG*parC*-AsyHRM method can detect the S83I variant quickly and directly and does not require routine interpretation (step 1 to step 3), which is particularly useful for the individualized treatment of M. genitalium. In previous research, 97% (166 of 171) of M. genitalium infections without an S83I mutation were cured, demonstrating the predictive value of S83I in clinical care (11). The high accuracy (98.7%) of our method in clinical samples (genomic copies ≥ 20 copies per reaction) meets the diagnostic needs of public health and clinical settings. Additionally, the sensitivity of common mutations (WT, S83N, S83I, S83R, D87Y, and D87N) was as low as 10 copies per reaction in the evaluation with a plasmid. We expect the method to maintain stable and accurate performance when there are ≥20 genomic copies per reaction in the clinical samples, which is significantly less than the infection load of clinical specimens of M. genitalium (5.50 × 10^3^ genomes/mL) (26). Notably, assay 1 can be used independently for routine identification of M. genitalium. M. genitalium diagnostics are insufficient in many settings, which underlies the need for the development of commercial kits (38). If the local AMR testing only requires the detection of specific mutations instead of a comprehensive screen, the *mgpa* target in assay 1 can be added directly in assay 2 to produce a local diagnostic method. The clinical specimens of M. genitalium infection are often coinfected with other STI-related pathogens, most commonly with Ureaplasma urealyticum, N. gonorrhoeae, and Chlamydia trachomatis. The results of cross-reactions show that our method works as intended, even with complex coinfections.

In addition, for species identification, the result of the two-way blind assessment of 184 samples showed 100% agreement between our method and the mass spectrum method (39). For AMR identification, the data demonstrated 100% consistency with Sanger sequencing. Importantly, 97/184 (52.7%) samples harbored coinfection (>2 STI-related agents), which further showed that this method can accurately detect M. genitalium in complex coinfection situations while also maintaining good performance. It is worth mentioning that all clinical samples underwent DNA purification using a common nucleic acid extraction kit instead of the special nucleic acid extraction kit for *Mycoplasma*. Additionally, nucleic acids of genitourinary normal flora were also included in the sample, and all 180 M. genitalium-negative samples (demonstrated by the mass spectrum method) showed no cross-reaction with the MG*parC*-AsyHRM assay.

This study highlights the great potential of the MG*parC*-AsyHRM method in the field of AMR testing; specifically, the type of approach taken in the MG*parC*-AsyHRM assay could be applied to different mutations and genes (Fig. 3). The target sequence in this study represents one of the most complex scenarios in AMR detection. Importantly, the application of an unlabeled probe coupled with HRM analysis produced an effective tool for AMR detection in genes with closely located AMR positions and a high diversity of mutations. This provides solutions for many existing problems of AMR detection, such as the closely located mutations on the *Mycoplasma pneumonia* 23S RNA gene (A2058G/C/T, A2059G, A2062G, and C2611A/G) (40). For culturable pathogens, the gene for species identification and the AMR gene can be directly combined into one assay, which can further improve the throughput of the method and lower costs. Another advantage of this method is the temperature adjustability, which provides the possibility for multiple detection and also for double probe detection. The diagnostic needs of public health and clinical settings are different, and the most common variant of the original probe can be easily changed to meet different detection needs.

One limitation in our method assessment is that clinical samples harboring rare mutations (S83C, D87G, D87H) were unavailable for our testing. However, all three of these variants are rarely reported, and a relation to fluoroquinolone resistance has not yet been proven (7, 33). According to the pairwise comparison of plasmids and clinical samples with other variants (such as S83I and S83R), we believe that the performance of the MG*parC*-AsyHRM method would not be affected by these rare variants.

In conclusion, this method can simultaneously distinguish eight variants related to fluoroquinolone resistance in the wild-type sequence and can detect M. genitalium. The Mg*parC*-AsyHRM method provides the advantages of high throughput, simple procedures, and low cost, demonstrating that our method can serve to enhance public health and the management of M. genitalium infections and AMR, providing a strong complement to phenotypic AMR surveillance to address the spread of fluoroquinolone resistance.

## MATERIALS AND METHODS

### Sample selection.

A total of 105 clinical samples, including 100 urethral swabs and 5 urine samples (male, 27; female, 78), were sourced from the Shenzhen Center for Chronic Disease Control. RT-PCR was used for species identification and to generate genomic copies of all samples (primers and probes are listed in Table S1 in the supplemental material, with details concerning the construction of the standard curve provided in the footnote). The *parC* locus was characterized using Sanger sequencing prior to performing the Mg*parC*-AsyHRM analysis (primers are listed in Table S2).

In addition, 184 clinical samples (urethral swabs) were collected from the Shenzhen Center for Chronic Disease Control for a two-way blind assessment of the Mg*parC*-AsyHRM method. All samples were processed using PCR coupled with a mass spectrum method previously reported for species identification (39).

An additional 33 samples were used to investigate the cross-reaction of the Mg*parC*-AsyHRM method with eight common STI-related pathogens, namely, Ureaplasma urealyticum (*n* = 5), Trichomonas vaginalis (*n* = 3), Chlamydia trachomatis (*n* = 5), Ureaplasma parvum (*n* = 5), Mycoplasma hominis (*n* = 5), Neisseria gonorrhoeae (*n* = 5), herpes simplex virus 1 (*n* = 2), and herpes simplex virus 2 (*n* = 3).

### Design of the Mg*parC*-AsyHRM method.

Fluoroquinolone resistance is closely associated with mutations of S83 and D87 in the *parC* allele. Mutations at both sites show high diversity, which poses difficulties for developing an AMR diagnostic method for M. genitalium. To combat this challenge, the Mg*parC*-AsyHRM method consists of two separate assays, assay 1 for species identification and nucleic acid quality control and assay 2 for M. genitalium AMR characterization. Assay 1 can also perform as an aid test for assay 2 to ensure the accuracy of the test results. For direct application on clinical samples, assay 1 was developed as a control test. The human β-globin (*HBB*) and *mgpa* allele were used as internal quality controls to ensure the performance of nucleic acid extraction and species identification, respectively. The *HBB* gene is widely recognized as an internal control gene in human samples that often coexists with target genes in clinical samples (41, 42). Because there are only slight differences in the *T_m_* value (<0.3°C) between mutations S83N and D87N in the combined products, using assay 2, it is difficult to quickly distinguish between these mutations on samples with poor quality (such as in cases with redundant salt ions and proteins in the sample). Therefore, paired primers in assay 1 flanking the mutation at the D87 site (G259) were used to correctly identify the S83N and D87N mutations according to the presence or absence of a mutation at D87. Assay 2 used one specific paired primer set and a 3′-blocked oligonucleotide probe. An 80-bp amplicon containing the S83 and D87 sites of *parC* allele was produced using the specific primer pair. However, due to inherent limitations of HRM, the method cannot distinguish between the same base mutation located at different sites, such as between S83I (G248T) and G87Y (G259T) or between S83N (G248A) and D87N (G259A). Consequently, the 32-bp oligonucleotide probe was designed to produce a short probe-amplicon. Because of the difference in sequence length, the *T_m_* of the probe-amplicon was significantly lower than that of the *parC*-amplicon. Furthermore, the short probe-amplicon sequences amplify the subtle differences between homogeneous mutations so that each mutation can be correctly distinguished. In this method, the probe is matched to the S83I variant, which is the most frequently detected mutation in the fluoroquinolone resistance-determining region (43). For all amplicons, the predicted *T_m_* value was evaluated using the online calculator OligoCalc (http://biotools.nubic.northwestern.edu/OligoCalc.html).

### Detection limit of the MG*parC*-AsyHRM method.

Nine plasmids containing various *parC* alleles (ParC WT, S83I, S83R, S83N, S83C, D87Y, D87N, D87G, and D87H) and one harboring the *mgpa* and *HBB* allele were used to determine the limit of detection (LOD) of the MG*parC*-AsyHRM method. All plasmids were serially diluted to 1000, 500, 200, 100, 50, 20, 10, and 2 copies/reaction. Each plasmid was tested at least 10 times to obtain a stable LOD value. The *parC* sequence of the plasmid (including wild type and mutant type) was in reference to the M. genitalium G37 isolate (GenBank accession number NC_000908).

### Flexibility of the MG*parC*-AsyHRM method.

We have designed five simple experiments to explore the flexibility of the MG*parC*-AsyHRM model by evaluating the following: (i) the effects of different probes, (ii) the performance of a probe harboring a double mutation (S83I plus D87Y), (iii) the compatibility of the probe with other genes, (iv) the generalizability of the MG*parC*-AsyHRM model, and (v) the adjustability of the MG*parC*-AsyHRM model.

### HRM procedures.

The RT-PCR assay was performed with a QuantStudio 6 Flex real-time PCR platform (Applied Biosciences, Foster City, CA, USA). Each sample contained 10 μL of EvaGreen master mix, 2 μL of DNA template, and the optimal concentration of primer as listed in Table 1, with double-distilled water (ddH_2_O) added to a final volume of 20 μL. The cycling conditions consisted of an initial hold for 10 min at 95°C, followed by 40 cycles of 95°C for 15 s and 60°C for 1 min. For the HRM analysis, the temperature was maintained at 40°C for 1 min and then slowly increased from 40°C to 95°C (0.025°C/s) for fluorescence collection (19).

### DNA extraction.

DNA purification of all clinical samples was performed on a MagNA Pure LC 2.0 instrument using the MagNA Pure LC nucleic acid isolation kit (Roche Diagnostics, USA) according to the manufacturer’s instructions. For urine, 1.5 mL of sample was extracted and eluted in 200 μL. The clinical samples were directly stored at −80°C before DNA extraction.

### Statistical analysis.

The *T_m_* values were calculated using SPSS software (v.21; SPSS Inc., Chicago, IL, USA) with 12 test replicates.

### Ethics statement

This study was approved by the Medical Ethics Committee at the Shenzhen Center for Chronic Disease Control (20180301). In accordance with the Helsinki Declaration, all participants’ personal data were anonymized in this study, and we obtained written informed consent for sample collection. The patents related to this article are pending (44). 

## References

[1] Daley GM, Russell DB, Tabrizi SN, McBride J. 2014. Mycoplasma genitalium: a review. Int J STD AIDS 25:475–487. doi:10.1177/0956462413515196. 24517928

[2] Kristiansen GQ, Lisby JG, Schonning K. 2016. A 5' nuclease genotyping assay for identification of macrolide-resistant Mycoplasma genitalium in clinical specimens. J Clin Microbiol 54:1593–1597. doi:10.1128/JCM.00012-16. 27053672 PMC4879279

[3] Jensen JS, Cusini M, Gomberg M, Moi H. 2016. 2016 European guideline on Mycoplasma genitalium infections. J Eur Acad Dermatol Venereol 30:1650–1656. doi:10.1111/jdv.13849. 27505296

[4] Bradshaw CS, Jensen JS, Waites KB. 2017. New horizons in Mycoplasma genitalium treatment. J Infect Dis 216:S412–S419. doi:10.1093/infdis/jix132. 28838073 PMC5853296

[5] Hamasuna R, Le PT, Kutsuna S, Furubayashi K, Matsumoto M, Ohmagari N, Fujimoto N, Matsumoto T, Jensen JS. 2018. Mutations in ParC and GyrA of moxifloxacin-resistant and susceptible Mycoplasma genitalium strains. PLoS One 13:e0198355. doi:10.1371/journal.pone.0198355. 29883482 PMC5993279

[6] Bachmann LH, Kirkcaldy RD, Geisler WM, Wiesenfeld HC, Manhart LE, Taylor SN, Seña AC, McNeil CJ, Newman L, Myler N, Fuchs R, Bowden KE, Danavall D, Morris M, Katz S, Nash E, Kersh E, Group MLW. 2020. Prevalence of Mycoplasma genitalium infection, antimicrobial resistance mutations, and symptom resolution following treatment of urethritis. Clin Infect Dis 71:e624–e632. doi:10.1093/cid/ciaa293. 32185385 PMC7744987

[7] Machalek DA, Tao Y, Shilling H, Jensen JS, Unemo M, Murray G, Chow EPF, Low N, Garland SM, Vodstrcil LA, Fairley CK, Hocking JS, Zhang L, Bradshaw CS. 2020. Prevalence of mutations associated with resistance to macrolides and fluoroquinolones in Mycoplasma genitalium: a systematic review and meta-analysis. Lancet Infect Dis 20:1302–1314. doi:10.1016/S1473-3099(20)30154-7. 32622378

[8] van der Schalk TE, Braam JF, Kusters JG. 2020. Molecular basis of antimicrobial resistance in Mycoplasma genitalium. Int J Antimicrob Agents 55:105911. doi:10.1016/j.ijantimicag.2020.105911. 31991219

[9] Gaydos CA. 2017. Mycoplasma genitalium: accurate diagnosis is necessary for adequate treatment. J Infect Dis 216:S406–S411. doi:10.1093/infdis/jix104. 28838072 PMC5853520

[10] Fernandez-Huerta M, Bodiyabadu K, Esperalba J, Bradshaw CS, Serra-Pladevall J, Garland SM, Fernandez-Naval C, Jensen JS, Pumarola T, Ebeyan S, Lundgren M, Tan LY, Espasa M, Murray GL. 2019. Multicenter clinical evaluation of a novel multiplex real-time PCR (qPCR) assay for detection of fluoroquinolone resistance in Mycoplasma genitalium. J Clin Microbiol 57:e00886-19. doi:10.1128/JCM.00886-19. 31434719 PMC6812999

[11] Sweeney EL, Bradshaw CS, Murray GL, Whiley DM. 2022. Individualised treatment of Mycoplasma genitalium infection-incorporation of fluoroquinolone resistance testing into clinical care. Lancet Infect Dis doi:10.1016/S1473-3099(21)00629-0. 35325618

[12] Bodiyabadu K, Danielewski J, Garland SM, Machalek DA, Bradshaw CS, Birnie J, Ebeyan S, Lundgren M, Murray G. 2021. Detection of parC gene mutations associated with quinolone resistance in Mycoplasma genitalium: evaluation of a multiplex real-time PCR assay. J Med Microbiol 70:001257. doi:10.1099/jmm.0.001257. 33612146 PMC8346731

[13] Le Roy C, Bebear C, Pereyre S. 2020. Clinical evaluation of three commercial PCR assays for the detection of macrolide resistance in Mycoplasma genitalium. J Clin Microbiol 58:e01478-19. doi:10.1128/JCM.01478-19. 31801835 PMC6989056

[14] Le Roy C, Bebear C, Pereyre S. 2021. Performance of three commercial molecular diagnostic assays for the simultaneous detection of Mycoplasma genitalium and macrolide resistance. J Clin Microbiol 59:e00020-21. doi:10.1128/JCM.00020-21. 33731412 PMC8316089

[15] Sweeney EL, Lowry K, Ebeyan S, Lundgren M, Whiley DM. 2020. Evaluation of the SpeeDx MG parC (beta) PCR assay for rapid detection of Mycoplasma genitalium quinolone resistance-associated mutations. J Clin Microbiol 58:e01432-20. doi:10.1128/JCM.01432-20. 32719034 PMC7512155

[16] Sweeney EL, Mhango LP, Ebeyan S, Tan LY, Bletchly C, Nimmo GR, Whiley DM. 2020. Evaluation of the ResistancePlus MG FleXible cartridge for near-point-of-care testing of Mycoplasma genitalium and associated macrolide resistance mutations. J Clin Microbiol 58:e01897-19. doi:10.1128/JCM.01897-19. 31896664 PMC7041587

[17] Harrison LB, Hanson ND. 2017. High-resolution melting analysis for rapid detection of sequence type 131 Escherichia coli. Antimicrob Agents Chemother 61:e00265-17. doi:10.1128/AAC.00265-17. 28416542 PMC5444143

[18] Golparian D, Dona V, Sanchez-Buso L, Foerster S, Harris S, Endimiani A, Low N, Unemo M. 2018. Antimicrobial resistance prediction and phylogenetic analysis of Neisseria gonorrhoeae isolates using the Oxford Nanopore MinION sequencer. Sci Rep 8:17596. doi:10.1038/s41598-018-35750-4. 30514867 PMC6279828

[19] Xiu L, Li Y, Wang F, Zhang C, Li Y, Zeng Y, Yin Y, Peng J. 2020. Multiplex high-resolution melting assay for simultaneous identification of molecular markers associated with extended-spectrum cephalosporins and azithromycin resistance in Neisseria gonorrhoeae. J Mol Diagn 22:1344–1355. doi:10.1016/j.jmoldx.2020.08.003. 32818599

[20] Xiu L, Li Y, Zhang C, Li Y, Zeng Y, Wang F, Peng J. 2020. A molecular screening assay to identify Chlamydia trachomatis and distinguish new variants of C. trachomatis from wild-type. Microb Biotechnol 14:668–676. doi:10.1111/1751-7915.13724. 33277967 PMC7936308

[21] Xiu L, Zhang C, Li Y, Wang F, Peng J. 2020. High-resolution melting analysis for rapid detection of the internationally spreading ceftriaxone-resistant Neisseria gonorrhoeae FC428 clone. J Antimicrob Chemother 75:106–109. doi:10.1093/jac/dkz395. 31834402

[22] Li Y, Zhang L, Xiu L, Wang D, Zeng Y, Wang F, Yin Y, Peng J. 2022. A multiplex molecular assay for detection of six penA codons to predict decreased susceptibility to cephalosporins in Neisseria gonorrhoeae. Antimicrob Agents Chemother 66:e01709-21. doi:10.1128/aac.01709-21. 35007131 PMC8923170

[23] Tindall EA, Petersen DC, Woodbridge P, Schipany K, Hayes VM. 2009. Assessing high-resolution melt curve analysis for accurate detection of gene variants in complex DNA fragments. Hum Mutat 30:876–883. doi:10.1002/humu.20919. 19280649

[24] Wu Z, Yuan H, Zhang X, Liu W, Xu J, Zhang W, Guan M. 2011. Development and inter-laboratory validation of unlabeled probe melting curve analysis for detection of JAK2 V617F mutation in polycythemia vera. PLoS One 6:e26534. doi:10.1371/journal.pone.0026534. 22028900 PMC3197667

[25] Lee HK, Lee CK, Loh TP, Tang JW, Tambyah PA, Koay ES. 2011. High-resolution melting approach to efficient identification and quantification of H275Y mutant influenza H1N1/2009 virus in mixed-virus-population samples. J Clin Microbiol 49:3555–3559. doi:10.1128/JCM.01087-11. 21865430 PMC3187298

[26] Murray GL, Danielewski J, Bodiyabadu K, Machalek DA, Bradshaw CS, Costa AM, Birnie J, Garland SM. 2019. Analysis of infection loads in Mycoplasma genitalium clinical specimens by use of a commercial diagnostic test. J Clin Microbiol 57:e00344-19. doi:10.1128/JCM.00344-19. 31243085 PMC6711907

[27] Jensen JS, Bradshaw CS, Tabrizi SN, Fairley CK, Hamasuna R. 2008. Azithromycin treatment failure in Mycoplasma genitalium-positive patients with nongonococcal urethritis is associated with induced macrolide resistance. Clin Infect Dis 47:1546–1553. doi:10.1086/593188. 18990060

[28] Hughes G, Saunders J. 2018. Mycoplasma genitalium: the next sexually transmitted superbug? BMJ 363:k4376. doi:10.1136/bmj.k4376. 30373885

[29] Hamasuna R, Osada Y, Jensen JS. 2007. Isolation of Mycoplasma genitalium from first-void urine specimens by coculture with Vero cells. J Clin Microbiol 45:847–850. doi:10.1128/JCM.02056-06. 17251394 PMC1829085

[30] Unemo M, Del Rio C, Shafer WM. 2016. Antimicrobial resistance expressed by Neisseria gonorrhoeae: a major global public health problem in the 21st century. Microbiol Spectr 4. doi:10.1128/microbiolspec.EI10-0009-2015. PMC4920088 27337478

[31] Montgomery J, Wittwer CT, Palais R, Zhou L. 2007. Simultaneous mutation scanning and genotyping by high-resolution DNA melting analysis. Nat Protoc 2:59–66. doi:10.1038/nprot.2007.10. 17401339

[32] Zaw MT, Emran NA, Lin Z. 2018. Mutations inside rifampicin-resistance determining region of rpoB gene associated with rifampicin-resistance in Mycobacterium tuberculosis. J Infect Public Health 11:605–610. doi:10.1016/j.jiph.2018.04.005. 29706316

[33] Li Y, Su X, Le W, Li S, Yang Z, Chaisson C, Madico G, Gong X, Reed GW, Wang B, Rice PA. 2020. Mycoplasma genitalium in symptomatic male urethritis: macrolide use is associated with increased resistance. Clin Infect Dis 70:805–810. doi:10.1093/cid/ciz294. 30972419 PMC7390511

[34] Wold C, Sorthe J, Hartgill U, Olsen AO, Moghaddam A, Reinton N. 2015. Identification of macrolide-resistant Mycoplasma genitalium using real-time PCR. J Eur Acad Dermatol Venereol 29:1616–1620. doi:10.1111/jdv.12963. 25622510

[35] Salado-Rasmussen K, Jensen JS. 2014. Mycoplasma genitalium testing pattern and macrolide resistance: a Danish nationwide retrospective survey. Clin Infect Dis 59:24–30. doi:10.1093/cid/ciu217. 24729494 PMC4305131

[36] Tickner JA, Bradshaw CS, Murray GL, Whiley DM, Sweeney EL. 2022. Novel probe-based melting curve assays for the characterization of fluoroquinolone resistance in Mycoplasma genitalium. J Antimicrob Chemother 77:1592–1599. doi:10.1093/jac/dkac097. 35352120 PMC9155627

[37] Fernandez-Huerta M, Esperalba J, Serra-Pladevall J, Espasa M. 2020. Mycoplasma genitalium and fluoroquinolone resistance detection using a novel qPCR assay in Barcelona, Spain. Enferm Infecc Microbiol Clin (Engl Ed) 38:293–294. doi:10.1016/j.eimc.2019.10.003. 31767219

[38] Naidu P, Shokoples S, Martin I, Zelyas N, Singh A. 2021. Evaluation of 5 commercial assays for the detection of Mycoplasma genitalium and other urogenital mycoplasmas. Med Microbiol Immunol 210:73–80. doi:10.1007/s00430-021-00699-1. 33595707

[39] Xiu L, Zhang C, Li Y, Wang F, Peng J. 2019. Simultaneous detection of eleven sexually transmitted agents using multiplexed PCR coupled with MALDI-TOF analysis. Infect Drug Resist 12:2671–2682. doi:10.2147/IDR.S219580. 31695443 PMC6717854

[40] Fyfe C, Grossman TH, Kerstein K, Sutcliffe J. 2016. Resistance to macrolide antibiotics in public health pathogens. Cold Spring Harb Perspect Med 6:a025395. doi:10.1101/cshperspect.a025395. 27527699 PMC5046686

[41] Zhang C, Xiu L, Xiao Y, Xie Z, Ren L, Peng J. 2018. Simultaneous detection of key bacterial pathogens related to pneumonia and meningitis using multiplex PCR coupled with mass spectrometry. Front Cell Infect Microbiol 8:107. doi:10.3389/fcimb.2018.00107. 29675400 PMC5895723

[42] Teblick L, Van Keer S, De Smet A, Van Damme P, Laeremans M, Rios Cortes A, Beyers K, Vankerckhoven V, Matheeussen V, Mandersloot R, Floore A, Meijer C, Steenbergen RDM, Vorsters A. 2021. Impact of collection volume and DNA extraction method on the detection of biomarkers and HPV DNA in first-void urine. Molecules 26:1989. doi:10.3390/molecules26071989. 33915837 PMC8036936

[43] Plummer EL, Murray GL, Bodiyabadu K, Su J, Garland SM, Bradshaw CS, Read TRH, Tabrizi SN, Danielewski JA. 2020. A custom amplicon sequencing approach to detect resistance associated mutations and sequence types in Mycoplasma genitalium. J Microbiol Methods 179:106089. doi:10.1016/j.mimet.2020.106089. 33184030

[44] Junping P, Yamei L. April, 2022. A molecular assay and kit for detection of parC mutation types in *Mycoplasma genitalium*. China patent application 202210393576.3.

