# Supplementary material for: Rapid Detection of Antimicrobial Resistance in Mycoplasma genitalium by High-Resolution Melting Analysis with Unlabeled Probes
Source: Microbiol Spectr. 2022 Jul 26;10(4):e01014-22. doi: 10.1128/spectrum.01014-22 (PMC9430336; doi:10.1128/spectrum.01014-22)
Supplement: Supplemental file 1 — Supplemental material. Download spectrum.01014-22-s0001.pdf, PDF file, 0.2 MB [file spectrum.01014-22-s0001.pdf]

Supporting Information

Legends of figures

Figure 1. Workflow of the MG*parC*-AsyHRM method
WT: Wild-type

Figure 2. Results of assays 1 and 2 of the MG*parC*-AsyHRM method

Figure 3. Flexibility of the MG*parC*-AsyHRM method. (a) Effect of a different probe; (b) performance of a probe harboring double mutations (S83I+D87Y); (c) compatibility of the probe with other genes; (d) generalizability of the MG*parC*-AsyHRM model; (e) adjustability of the MG*parC*-AsyHRM model.

Supplementary information

Table S1 Primers and probes of real-time PCR used in this method.

Table S2 Primers of common PCR used in this study.

Table S3 Limit of detection of each target variant.

Figure S1 Illustration of sequence structure from 83 to 87 position in *parC* gene.

Table S1. Primers and probes of real-time PCR used in this method.

Target gene	Direction	Primers	Reference
MGpa	Forward	GAGAAATACCTTGATGGTCAGCAA	(1)
	Reverse	GTTAATATCATATAAAGCTCTACCGTTGTTATC	
	probe	FAM-ACTTTGCAATCAGAAGGT-MGB	

standard curve method:

We prepared plasmid containing *porA* and was diluted into a series of gradients (range from 1copy/ μ L to 1 x 10^4copy/ μ L) for making standard curve. The procedure of quantification is the same as described previously (2).

Table S2 Primers of common PCR used in this study

Target gene	Direction	Primers	Size (bp)	Reference
parC	in-Forward-119F1	5'GGTTAAAACCAGTACAAAGACGGA3'	226	this study
	in-Reverse-385R1	5'ACACAGAAACCCGCTTAAGCT3'		
	out- Forward -97F2	5'GCTTTACCTGATCTAAGAGATGGGT3'	427	
	out- Reverse--524R2	5'GCTATCCCACTCGCACCATT3'		

Table S3, Limit of detection of each target variant.

Target	No. of replicates (%) for positive standards, copies/reaction															
	1000		500		200		100		50		20		10		2	
Assay2	probe[*]	*parC*[#]	probe	*parC*	probe	*parC*	probe	*parC*	probe	*parC*	probe	*parC*	probe	*parC*	probe	*parC*
WT	10/10	10/10	10/10	10/10	10/10	10/10	10/10	10/10	10/10	10/10	10/10	10/10	10/10	10/10	1/10	8/10
S83C	10/10	10/10	10/10	10/10	10/10	10/10	10/10	10/10	10/10	10/10	10/10	10/10	9/10	10/10	1/10	10/10
S83N	10/10	10/10	10/10	10/10	10/10	10/10	10/10	10/10	10/10	10/10	10/10	10/10	10/10	10/10	2/10	10/10
S83R	10/10	10/10	10/10	10/10	10/10	10/10	10/10	10/10	10/10	10/10	10/10	10/10	10/10	10/10	2/10	9/10
S83I	10/10	10/10	10/10	10/10	10/10	10/10	10/10	10/10	10/10	10/10	10/10	10/10	10/10	10/10	2/10	8/10
D87H	10/10	10/10	10/10	10/10	10/10	10/10	10/10	10/10	10/10	10/10	10/10	10/10	6/10	10/10	0/10	5/10
D87Y	10/10	10/10	10/10	10/10	10/10	10/10	10/10	10/10	10/10	10/10	10/10	10/10	10/10	10/10	10/10	5/10
D87N	10/10	10/10	10/10	10/10	10/10	10/10	10/10	10/10	10/10	10/10	10/10	10/10	10/10	10/10	1/10	10/10
D87G	10/10	10/10	10/10	10/10	10/10	10/10	10/10	10/10	10/10	10/10	10/10	10/10	3/10	10/10	0/10	7/10
Assay1																
D87MT	10/10		10/10		10/10		10/10		10/10		10/10		10/10		9/10	
D87WT	10/10		10/10		10/10		10/10		10/10		10/10		10/10		7/10	
mgpa	10/10		10/10		10/10		10/10		10/10		10/10		10/10		4/10	
HBB	10/10		10/10		10/10		10/10		10/10		10/10		10/10		9/10	

[*]probe，probe-amplicon; [#]*parC*, *parC*-amplicon

1、Jensen JS, Björnelius E, Dohn B, Lidbrink P. Use of TaqMan 5' nuclease real-time PCR for quantitative detection of Mycoplasma genitalium DNA in males with and without urethritis who were attendees at a sexually transmitted disease clinic. J Clin Microbiol. 2004 Feb;42(2):683-92. doi: 10.1128/JCM.42.2.683-692.2004. PMID: 14766837; PMCID: PMC344445.

2、 Dupin N, Bijaoui G, Schwarzinger M et al. Detection and quantification of *Mycoplasma genitalium* in male patients with urethritis. Clin Infect Dis. 2003 Aug 15;37(4):602-5. doi: 10.1086/376990. Epub 2003 Jul 30.

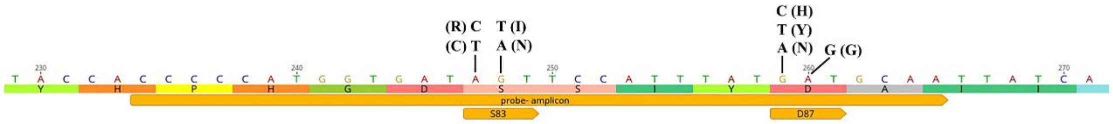

Figure S1. Illustration of sequence structure from 83 to 87 position in *parC* gene.
